# A.A.A. Good Wines WANTED: Blockchain, Non-Destructive Ultrasonic Techniques and Soil Health Assessment for Wine Traceability

**DOI:** 10.3390/s25113567

**Published:** 2025-06-05

**Authors:** Diego Romano Perinelli, Martina Coletta, Beatrice Sabbatini, Aldo D’Alessandro, Fabio Fabiani, Andrea Passacantando, Giulia Bonacucina, Antonietta La Terza

**Affiliations:** 1 School of Pharmacy, University of Camerino, Via Madonna delle Carceri 9, 62032 Camerino, MC, Italy; diego.perinelli@unicam.it (D.R.P.); beatrice.sabbatini@unicam.it (B.S.); 2 School of Biosciences and Veterinary Medicine, University of Camerino, Via Gentile III da Varano, 62032 Camerino, MC, Italy; martina.coletta@unicam.it (M.C.); aldo.dalessandro@unicam.it (A.D.); 3 EGG CHAIN S.r.l., Via Le Piane 23B, 62036 Pieve Torina, MC, Italy; fabio.fabiani@eggchain.net; 4 Azienda Agricola Passacantando Andrea, C. da Ributino 7, 62029 Tolentino, MC, Italy; andrea@passacantando.it

**Keywords:** QBS-ar, sound speed, vineyards, wine cellar, biodiversity, sustainability, viscosity, density

## Abstract

The wine industry faces increasing challenges related to authenticity, safety, and sustainability due to recurrent fraud, shifting consumer preferences, and environmental concerns. In this study, as part of the B.I.O.C.E.R.T.O project, we integrated blockchain technology with ultrasonic spectroscopy and soil quality data by using the arthropod-based Soil Biological Quality Index (QBS-ar) to enhance traceability, ensure wine quality, and certify sustainable vineyard practices. Four representative wines from the Marche region (Sangiovese, Maceratino, and two Verdicchio PDO varieties) were analyzed across two vintages (2021 and 2022). Ultrasound spectroscopy demonstrated high sensitivity in distinguishing wines based on ethanol and sugar content, comparably to conventional viscosity-based methods. The QBS-ar index was applied to investigate the soil biodiversity status according to the agricultural management practices applied in each vineyard, reinforcing consumer confidence in environmentally responsible viticulture. By recording these data on a public blockchain, we developed a secure, transparent, and immutable certification system to verify the geographical origin of wines along with their unique characteristics. This is the first study to integrate advanced analytical techniques with blockchain technology for wine traceability, simultaneously addressing counterfeiting, consumer demand for transparency, and biodiversity preservation. Our findings support the applicability of this model to other agri-food sectors, with potential for expansion through additional analytical techniques, such as isotopic analysis and further agroecosystem sustainability indicators.

## 1. Introduction

According to the Assoenologi Observatory, Ismea, and the Italian Wine Union (UIV), the grape harvest for 2024 is estimated to be 41 million hectolitres, which represents a 7% increase compared to 2023. Although this volume is lower (−12.8%) than the average of the last five years, Italy confirms its role as the world’s leading wine producer, especially in light of the sharp decline in French production [1]. Nonetheless, a slowdown in global demand was registered, likely due to uncertainties in the economic and geopolitical landscape, as well as shifts in consumption patterns, especially among younger generations. Emerging trends in wine consumption include a heightened emphasis on health, safety, and the environmental impact of food production [1].

A recent study found that 68% of Italians are concerned about the impact of their dietary choices on their health [2]. This concern stems partly from repeated food scandals. A notable case is the 2018 fraud, where 150,000 L of generic white wine was fraudulently sold as Verdicchio dei Castelli di Jesi PDO (Protected Designation Origin) [3]. This case exemplifies how current traceability systems are vulnerable to falsification, particularly in certifying authentic geographical origin and production practices. At the European level, wine represents a major agricultural export. However, it is also one of the most frequently counterfeited food products [4], with fraudulent practices in the sector estimated to cost around 1.3 billion euros annually [5]. This situation underscores the limitations of current PDO controls, which rely mainly on (a) document-based traceability (paper or centralized databases) and (b) certification of origin and production methods based largely on self-declarations by producers and regulatory inspections. These systems fall short because they (a) are vulnerable to document manipulation; (b) lack integration with independently verifiable scientific data; (c) cannot uniquely authenticate the chemical–physical identity of a specific wine; and (d) do not assess environmental sustainability or biodiversity-friendly practices. As a result, the current PDO framework is unable to provide consumers with a robust, tamper-proof guarantee of a wine’s origin, quality, and sustainable production.

In this regard, recurring and patterned frauds have not only discouraged consumers, but have also undermined their confidence in wine products. The primary concerns among consumers revolve around the authenticity, safety, and quality of wines. So far, various methodological approaches and accurate characterization methods to ensure authenticity and market transparency have been identified to meet this demand.

Among these, blockchain technology is certainly central. Blockchain technology is a decentralized ledger containing information on a product or process recorded in a distributed peer-to-peer network that guarantees its security, traceability, immutability, non-clonability, transparency, and accessibility, while respecting privacy. With such technology, it is possible to register and notarize on a public register (public blockchain) the unique characteristic information of an agri-food product with a certain date (i.e., wine) to certify the geographical origin through an infallible cryptographic system that cannot be modified externally.

To date, blockchain technology has been applied to a wide range of agri-food products, from extra virgin olive oil (E.V.O.O.) to various types of cereals, among others. In particular, for these latter categories, blockchain has been integrated with complementary technologies—such as the Internet of Things (IoT) in the case of E.V.O. [6,7,8], and the Acceptance Model for ancient grain varieties [9,10], with the objective of enhancing transparency, traceability, and overall data integrity across the entire agri-food supply chain.

The use of blockchain technology for wine traceability requires the collection of chemical–physical characteristics that can be unequivocally attributed to each vineyard and relative wine produced. Several traditional techniques are currently available for wine characterization, including sensory analysis, chromatography, rheology, and spectrophotometry [11]. However, ultrasonic spectroscopy has recently emerged as a new easily applicable technique [11,12,13] for the chemical–physical characterization of food and beverages, including wine, in a non-invasive, non-destructive, and easily automated manner [14,15]. Ultrasounds are acoustic waves with a frequency above the audible range for humans (~20 kHz), which can be generated from piezoelectric elements and propagated longitudinally through a material via adiabatic compression and decompression cycles [16,17]. Low-intensity and high-frequency ultrasounds (frequency above 100 kHz) can be experimentally employed to investigate the chemical–physical properties of materials by monitoring the variation in ultrasound wave parameters, such as the sound speed and attenuation, which are dependent on the structure of the material itself [18]. Different high-frequency ultrasound devices, operating in single-frequency, frequency scanning, or simultaneous multi-frequency scanning modes have been employed for the characterization of the chemical–physical properties of a large variety of monophasic, heterogeneous, or colloidal materials, including paints, cosmetics, ceramics, foods, and pharmaceutical products [12,19,20].

With regard to the characterization of wines, the main application of techniques employing high-frequency ultrasounds has been the real-time monitoring of the wine fermentation process [13]. Novoa-Diaz et al. monitored the conversion of malic acid into lactic acid during the fermentation process in red wines using 1 MHz ultrasound longitudinal waves [21]. Similarly, Lamberti et al. investigated the alcoholic fermentation process of wine in comparison to saccharose–ethanol model solutions, using a similar ultrasound apparatus [22]. Only more recently, high-frequency ultrasound spectroscopy has been proposed as a tool to discriminate between white and red wines as well as among different varieties [11].

Apart from aspects relating to the chemical and physical characteristics of the wine and its safety, consumers are increasingly interested in environmental issues, preferring wines produced using sustainable agricultural practices that respect biodiversity [1]. A choice experiment on wine consumers estimated their willingness to pay for biodiversity conservation practices in vineyards, confirming their interest in this topic [23]. Adopting sustainable soil management practices in vineyards can help maintain the ecological balance and enhance vine health. Additionally, understanding the relationship between vineyard management and soil biodiversity is crucial for achieving environmentally and economically sustainable viticulture [24]. To evaluate the sustainability of the agricultural practices applied in vineyards, various bioindicators can be used. One of the most commonly used metrics to assess the impact of agricultural practices on soil biodiversity is the arthropod-based Soil Biological Quality Index (QBS-ar) [25,26]. This index has already been successfully used to assess soil quality in vineyards [24,27,28,29,30,31].

In the present study, as part of the project B.I.O.C.E.R.T.O (Marche region, RDP 2014/22-M 16.1, ID 41377), we propose an integrated tri-modal workflow that combines ultrasound spectroscopy, the QBS-ar index, and blockchain technology. All together, these three methodologies form a scientifically robust, technologically advanced, and consumer-transparent traceability system, capable of (1) certifying wine authenticity through objective physical–chemical data (i.e., ultrasonic spectroscopy); (2) certifying the use of sustainable agricultural practices in vineyards, promoting soil biodiversity conservation by using the QBS-ar index; and (3) preventing fraud through immutable, distributed data storage (i.e., blockchain).

To achieve this, four wine samples representative of the Marche region were selected. These included one red wine (Sangiovese) and three white wines: Maceratino and two varieties of Verdicchio, representing two distinct PDO areas, Matelica and Castelli di Jesi (Table 1). It is important to emphasize the territorial relevance of the selected grape varieties. The two Verdicchio samples represent the flagship white wine of the Marche region, known for its historical and enological importance. By including both Verdicchio di Matelica and Verdicchio dei Castelli di Jesi, the study captures the diversity within this varietal, as shaped by different microclimates and terroirs. Maceratino is an indigenous grape variety native to the Marche region, which has been recently rediscovered and is currently produced in limited quantities, particularly for single-varietal winemaking. As for Sangiovese, although it is not the most widely cultivated grape variety in the region (especially when compared to Verdicchio), it is used to produce high-quality red wines under the “Marche Sangiovese IGT” (Indicazione Geografica Tipica—Typical Geographical Indication) designation. Its inclusion ensures representation of the region’s red wine potential and aligns with the aim of covering different typologies and denominations within the study. The wines were analyzed for two consecutive vintages (2021 and 2022). In addition, the QBS-ar index was applied for two years to the vineyards from which the wines originated.

Although blockchain has been widely applied in the wine industry to trace origin and supply chain steps [32], this study is the first to integrate ultrasound spectroscopy and the QBS-ar index into a blockchain system alongside traditional data. The proposed tri-modal pilot approach has the potential to enhance traceability by certifying authenticity, chemical–physical identity, and environmental sustainability, thereby addressing key gaps in current PDO systems. It offers a robust, transparent, and science-based certification model, reinforcing the value of *Made in Italy* wines and restoring consumer confidence.

## 2. Materials and Methods

### 2.1. Study Sites: Vineyards and Wines

**Figure 1:**
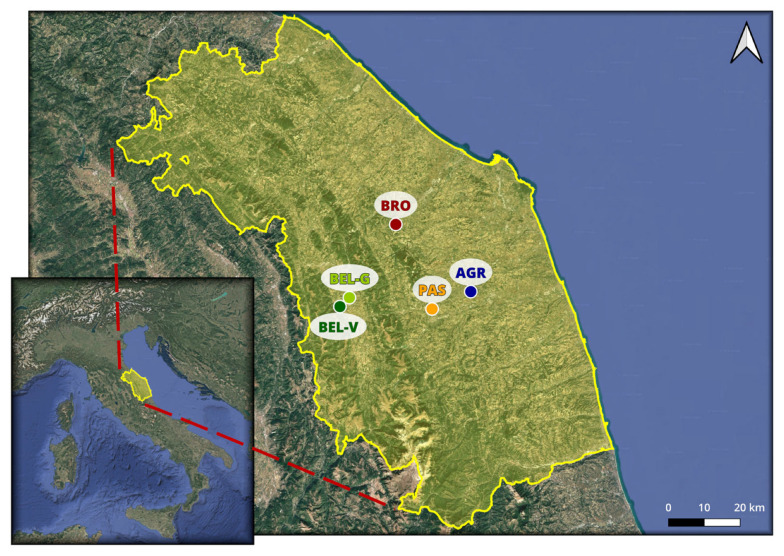
Map of Italy and distribution of the study sites in the Marche region. The map was made with QGIS v. 3.40.5 LTR software. The labels indicate the locations of the various farms and vineyards, based on the vineyard ID listed in Table 1.

The farms involved in the project and the type of wines, analyzed using non-destructive ultrasonic techniques, are shown in Table 1. All participating wineries were organically managed. Unfortunately, due to production problems, the Maceratino wine (AGR) was only analyzed for a single vintage (2021). For the Belisario winery, due to the extensive size of the vineyard (about 20 hectares) and the different planting ages (BEL-G, BEL-V), it was deemed necessary to collect two QBS-ar samples to ensure more reliable data (see Figure 1).

Az. Agr. Istituto Statale di Istruzione Superiore (I.I.S.) G. Garibaldi

The I.I.S. G. Garibaldi in Macerata has a rich history that dates back to 1868. The Agricultural School features a farm that covers over 70 hectares. The grape varieties grown there include Sangiovese, Montepulciano, Ciliegiolo, Maceratino, Merlot, Lacrima, Verdicchio, Fiano, and Grechetto. The wines produced at the Institute include Rosso Piceno PDO, Colli Maceratesi Bianco PDO (Ribona), and rosé, white, and red table wine. The school has a vital role in education, serving as an essential resource for the practical application and development of specific skills in the agricultural sector.

Az. Agr. Brocani

The Brocani farm is a family-run winery that exclusively produces and vinifies grapes from its own organic vineyards, which are located across the municipalities of Staffolo, Cupramontana, and San Paolo di Jesi. The farm covers approximately 12.5 hectares and transforms all its production into a variety of wines, including Verdicchio Classico, Esino Rosso PDO, Vino Rosso IGT, Vino Bianco IGT, and flavored wines. The grape varieties grown there include Verdicchio and Montepulciano. The winery focuses on high-quality production while promoting environmental sustainability, regional connections, authenticity, and more mindful consumption.

Az. Agr. Passacantando

The Passacantando farm was established in 1996, building on a family farming tradition that began in the 1960s. The farm covers 90 ha across various locations in the municipalities of Tolentino and San Severino Marche, where they cultivate cereals, forage, and olives. Additionally, the farm has 2 hectares of vineyards, specifically Sangiovese. Currently, the farm is managed organically and has diversified its activities to include agritourism.

Cantina Belisario

Cantina Belisario is a 300-hectare vineyard and the largest producer of Verdicchio di Matelica PDO, a typical white wine from the Marche region. All the vineyards are located in the Alta Valle Esina. The company manages the vineyards directly, with each wine label linked to a specific vineyard. Each vineyard represents a distinct project that begins with the initial planting. The careful selection of grapes and musts is aimed at achieving the highest quality to meet the needs and expectations of Belisario wine purchasers.

### 2.2. Pedological and Climatic Characteristics of the Study Area

The study focused on soil provinces 3.3 and 5.3, which are neighboring areas located in the central part of the Marche region, mainly covering the provinces of Ancona and Macerata. (Table 2). In the 3.3 province, the landscape features undulating slopes with generally low gradients and terraced plains that rise between approximately 200 and 950 masl. The geological substrate includes marls, arenitic–pelitic materials, and calcarenitic deposits from the minor basins of the Marches, as well as Pleistocene and Holocene alluvium [33]. In the 5.3 province, the topography consists of slopes and plateaus with medium to low gradients and some areas of desiccation, situated between roughly 150 and 600 masl. The underlying materials here are primarily Pliocene and Pleistocene marls and clays, along with foothill alluvium [33]. Both provinces have extensive areas dedicated to wine growing. In all cases, the soils considered were neutral (pH 6.0–7.5).

To define the climate within the study area, meteorological data from the Agenzia per l’Innovazione nel Settore Agroalimentare e della Pesca (AMAP) (Jesi, Italy) were analyzed. The dataset included the monthly mean temperatures (°C) and precipitation (mm) recorded from January 2022 to December 2023 by the nearest meteorological stations, at similar altitudes to the sites considered.

### 2.3. Chemical–Physical Characterization of Wines

#### 2.3.1. Viscosity Measurements

Viscosity measurements were performed using a rotational rheometer (Kinexus Lab+, Malvern Instruments Limited, Malvern, Worcestershire, UK). Tests were carried out using a C40/4 cone-plate geometry at 25 °C in the shear rate range between 1 and 100 s^−1^. The viscosity values were calculated by fitting the shear stress vs. shear rate plots using a power law equation model:

$$Y=a\;X^{n}$$

where *Y* is the shear stress, X is the shear rate, a is the power law viscosity (Pa s), and n is the power law index. The data were the mean ± standard deviation of three independent measurements.

#### 2.3.2. High-Resolution Ultrasound Spectroscopy (HR-US) Analyses

High-resolution ultrasonic spectroscopy is an ultrasonic technique based on the resonance phenomenon between the sample and its medium as a reference. A transmitter generates a longitudinal ultrasonic wave, which travels through a sample cell and is reflected by a receiver, creating a stationary wave with amplified amplitude. The speed of the wave is calculated from its frequency and wavelength, while ultrasonic attenuation determines the half-bandwidth of resonant peaks, enabling measurements of velocity and attenuation. By analyzing resonant peaks across different frequencies (1–18 MHz), the frequency dependence of ultrasonic parameters can be investigated. The device uses two piezo transducers positioned opposite each other (the path length between transducers was approximately 1 cm), allowing measurements with high resolution (down to 0.2 mm/s for velocity) in a wide range of sample volumes. The standard setup includes a 2 mL thermostated cell (5–120 °C) with stirring capability, and supports various operating programs like temperature ramp, kinetic mode, titration mode, and multi-frequency mode [12,34].

HR-US analyses were performed using a high-resolution ultrasound spectrometer (HR-US 102; Ultrasonic Scientific, Dublin, Ireland). The instrument was fitted with two 2 mL-capacity cells: one for the sample and the other one for the reference. Water was employed as a reference liquid. The ultrasound parameters such as sound speed (m/s) and attenuation (1/m) were recorded for each sample of wine at a temperature of 25.00 ± 0.02 °C and a frequency of 5.4 MHz, selected after a broad frequency screening (1–16 MHz). The operational 5.4 MHz, being an intermediate resonant frequency, can ensure a good compromise between ultrasound penetration depth, spatial resolution, and sensitivity to the acoustic properties of the wine (Figure S1). The absolute ultrasound parameters were measured over a time of 300 s for each analysis and reported as the mean of the collected values. No filtration or degassing was performed prior to the analysis to preserve the original wine matrix. The data were the mean ± standard deviation of three independent measurements.

#### 2.3.3. Refractive Index and Density Measurements

Refractive index measurements were carried out at 25 °C using an Abbe Refractometer (NAR-1T LIQUID, Atago Co., LTD, Minato-ku, Tokyo, Japan), featuring the refractive index scale and the BRIX scale. Density measurements were carried out at 25 °C using a digital densimeter with an oscillating U-tube (DMA-5000M, Anton-Paar, Graz, Austria). The data are reported as the range of measurements of three independent measurements.

### 2.4. Soil Health Assessment Using the Arthropod-Based Biological Soil Quality Index (QBS-ar)

The arthropod-based Biological Soil Quality Index (QBS-ar) [25,26] was used to assess the soil health conditions of the various vineyards involved in the present study. The index is determined by identifying the main groups of soil microarthropods and assigning an Ecomorphological Index (EMI), ranging from 1 to 20, based on the microarthropod’s level of adaptation to soil life. The sum of the EMI provides the QBS-ar value, which indicates soil quality. At each vineyard, one QBS-ar sample, consisting of three subsamples of soil cores (A, B, C), was collected at a 10 cm depth. The subsamples were collected from the central area of the vineyard, spaced approximately 10 m apart in various inter-rows, and located away from the vineyard’s edge. A steel core drill (⌀ = 10 cm) was used to obtain an equivalent volume of soil, approximately 800 cm^3^. The samples were transported to the laboratory and, within 24 h, placed in a Berlese–Tullgren selector, equipped with 25 W light bulbs (210 lm) and a 2 mm sieve mesh for the extraction of soil microarthropods, which was completed over a period of 7 days. The total QBS-ar and partial QBS-ar values were calculated for each experimental site [27]. Once the QBS-ar value (both partial and total) was defined, it was compared with the numerical ranges reported in [35] for assigning the biological soil quality judgment. The numerical intervals vary depending on the management system considered. In this case, we specifically focused on tree crops and vineyards (whose value ranges from 61–80 = poor to >160 = excellent). Soil samples were collected twice from the same vineyards, in June 2022 and 2023. The partial QBS-ar data were subjected to the Shapiro–Wilk test for normality before analysis. The data were then compared between years (Wilcoxon–Mann–Whitney test) and between vineyards (Kruskal–Wallis test). In addition, differences between vineyards were assessed via post hoc test (Dunn test). A *p*-value < 0.05 was considered significant. Statistical analysis was performed using R software, version 4.2.0 [36].

### 2.5. The B.I.O.C.E.R.T.O. Blockchain Development

The digital platform (B.I.O.C.E.R.T.O.) consists of front-end and back-end microservices connected to a cloud data center, connected to a public blockchain network. For the Biocerto project, we have chosen to use the public blockchain “Quadrans” [37]. Quadrans is an open-source, public, and decentralized blockchain for storage and data sharing. Organizations around the world can use Quadrans to create cross-border automatisms to improve process execution and facilitate data management. The Quadrans team has developed a new consensus algorithm to improve the efficiency of the Quadrans blockchain; it uses both Proof of Stake and Proof of Work protocols and combines them into a modified Proof of Authority.

The B.I.O.C.E.R.T.O platform was developed within the project to analyze and manage all phases of an integrated production chain.

The information collected along the production chain was archived in the cloud on the platform and the digital files notarized on the public blockchain through complex cryptographic systems, to guarantee the immutability and unchangeability of the data and information collected, and to provide transparency on the production processes. Various processes were recorded on the platform, including the identification of the system and the census of the farms and sites involved in the study, the planning of the sampling calendar with the assignment of tasks, the execution of the sampling, the data on the collection of soil samples, and the related metadata.

For example, soil sampling events and soil and wine analysis output files were recorded, and the original files were uploaded to the digital platform. The terrain and corresponding coordinates were associated with each sample.

All this information is summarized in the form of a Biocerto Digital Certificate. At this point, the Quadrans blockchain API is invoked for the notarization of the original files and the Biocerto Digital Certificate of the product. QR codes are generated to be associated with the product and the territory of origin. The notarial register, the original files, and the platform can only be consulted by authorized users. The front-end of the platform is visible only under authentication for administrators’ use, while a public front-end visible to all consumer users is available, which allows them to view the Biocerto Digital Certificate using the QR code and the authenticity verification of the Certificate.

The prototype of the blockchain network represents the distributed digital infrastructure on which all Biocerto notarization services were based, and it is supported by the “Quadrans” public infrastructure.

To use these services, some computational nodes have been configured exposed on the external internet network capable of accessing the Quadrans API for notarizing the digital files collected.

The blockchain network consisted of physical servers (minicomputers) and virtual servers (cloud data centers) called nodes, with the specific hardware and software characteristics for the type of service required.

The registry of notarization was unique, distributed and shared among all the nodes of the network and was, by definition, decentralized, allowing for an additively scalable solution.

Regarding the operational costs of using the Biocerto blockchain, these include the purchase of computing nodes—specifically, a ‘master node’ computer for each ‘operator’ utilizing Biocerto and the Quadrans blockchain network. Each computer consumes approximately 200 watts of power per hour of operation.

## 3. Results and Discussion

### 3.1. Climatic Characteristics of the Study Area

**Figure 2:**
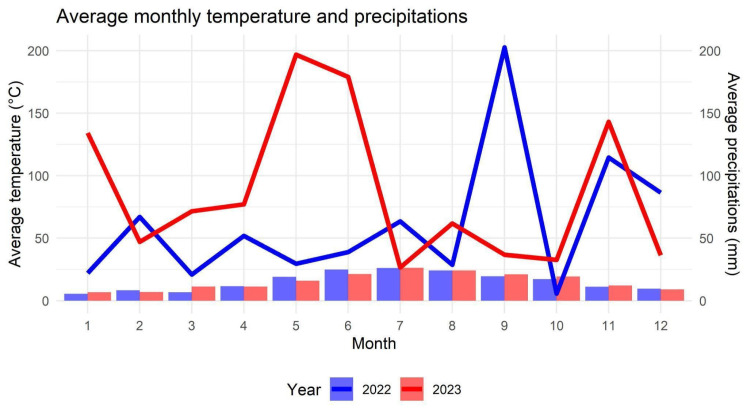
Graph of average temperature (°C) and precipitation (mm) in the years 2022 and 2023 obtained from 4 different meteorological stations, considering those closest to similar altitudes to the sites considered. The package “ggplot2” was used to generate the plot [36].

Considering the average monthly temperature, significant differences were found between years in March, May, June, September, and October (Figure 2; Table S1). Since the samplings were conducted in June, the variations observed in March and May may have had a more significant influence than the others. However, the overall differences in temperature were minimal and negligible.

Conversely, a distinct trend was observable between the two years for rainfall; the patterns in 2022 and 2023 were completely different (Figure 2; Table S1). Significant differences in precipitation were found in January, February, March, May, June, August, September, and December. While significant differences in precipitation were observed between years, it is hypothesized that the meteorological data had no effect on the biological data, as similar values were recorded across the years.

### 3.2. Viscosity Determination

**Figure 3:**
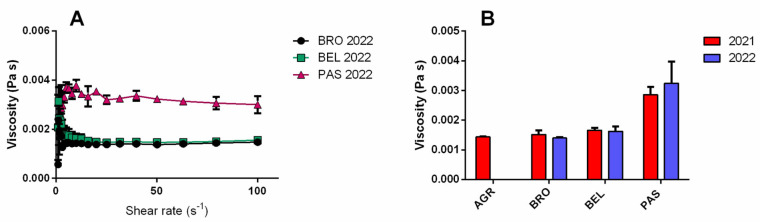
Viscosity (Pa s) vs. shear rate (s^−1^) plots for the analysis of wines (BRO, BEL, and PAS) produced in 2022 (**A**); comparison between viscosity (Pa s, Mean ± SD, n = 3) measured for the analyzed wines (AGR, BRO, BEL, and PAS) produced in 2021 and 2022 (**B**).

All the wines analyzed (AGR, BRO, PAS, BEL; Table 1) showed the typical behavior of newtonian liquids, in which the measured viscosity is independent of the shear rate applied, at least in the range investigated (1–100 s^−1^) (Figure 3A) [38]. The measured viscosity was in the range of 0.015–0.03 Pa s, with the highest value calculated for the PAS wine (0.028–0.030 Pa s). On the other hand, lower viscosity values (below 0.020 Pa s) were calculated for the other wines (AGR, BRO, and BEL). The calculated power law indexes were close to 1 for all wines in the range 0.972–1.054. No statistically significant differences were found between the viscosity values of the same wine produced in different years (2021 and 2022) (multiple *t*-test unpaired, statistical significance *p* < 0.05) (Figure 3B).

### 3.3. High-Resolution Ultrasound Characterization

Parameters from HR-US technology, such as sound speed (m/s) and attenuation (1/m) recorded at high-intensity and low-frequency (2–100 kHz) ultrasounds, can be employed to investigate the structural and chemical–physical properties of materials. When the ultrasound waves pass through a material, the velocity of propagation of the wave (sound speed) changes, and the wave itself loses part of its energy, leading to an increase in the attenuation parameter. Indeed, the propagation of the ultrasound waves in a material is dependent on the structure and composition of the materials, since they are affected by the nature of the chemical bonds and mass of molecules and/or supramolecular aggregates composing the sample. Therefore, HR-US configures as a versatile and non-destructive technique to characterize materials of a different nature by monitoring the differences and variations in ultrasound parameters. The HR-US technique can perform ultrasound measurements with a high resolution of up to 0.2 mm/s for sound speed and 0.2% for attenuation, being suitable for the analysis of liquid samples with a low concentration of solutes, such as wines.

The sound speed (*U*) depends on the density (*ρ*) and the adiabatic compressibility (*β*) of the liquid, according to the Laplace equation:

$$U=\frac{1}{\sqrt{\beta \rho }}$$

Ultrasonic attenuation (α) refers to the decrease in the fluctuation amplitude (*A*) during the travel of the ultrasound wave through the material, since a loss of energy transported by the wave occurs according to the following equation:

$$A=A_{0}\;e^{-\alpha z}$$

where *A*_0_ is the initial amplitude and *z* is the traveled distance.

**Figure 4:**
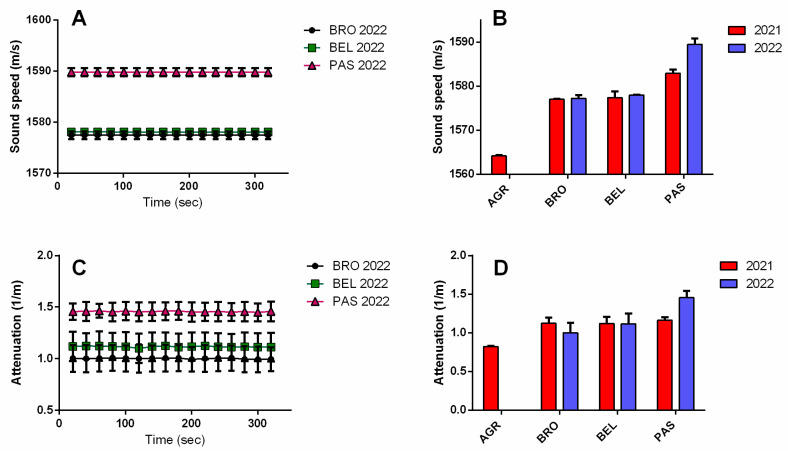
Sound speed (m/s) vs. time (s) (**A**) and attenuation (1/m) vs. time (**C**) plots for the analysis on wines (BRO, BEL, and PAS) produced in 2022; Comparison between sound speed (mean ± SD, n = 3) (**B**) and attenuation (mean ± SD, n = 3) (**D**) measured for the analyzed wines (AGR, BRO, BEL, and PAS) produced in 2021 and 2022.

Figure 4A,C report the raw data from sound speed attenuation over the measurement time (360 s) of the three wines, from which the mean ± SD values were calculated (Figure 4B,D). A similar trend with respect to viscosity was found for the ultrasound parameters. Specifically, PAS wine showed the highest sound speed values for both analyzed years (~1583 m/s for 2021 and ~1589 m/s for 2022) with respect to the other wines that displayed lower values (for BRO and BEL wines, the sound speed was between 1577 and 1579 m/s for both years, and for AGR, the sound speed was ~1565 m/s for 2021).

Yet, PAS wine showed the highest attenuation value, at least for the year 2022, but larger variability among measurements, resulting from the higher relative standard deviation (RSD %, range between 6 and 12) with respect to sound speed measurements (RDS% range between 0.01 and 0.04). This suggests that attenuation is a less accurate ultrasound parameter. This can be attributed to the higher instrumental noise sensitivity and complexity of the measurement for the attenuation parameter (e.g., adsorption, scattering phenomena of interaction with the ultrasound wave).

The three wines (BRO, BEL, and PAS) produced in 2022 and AGR wine produced in 2021 were further characterized in terms of the refractive index and density. Refractive index measurements provided the refractive index and Brix values listed in Table 3. From the Brix values, representing the grams of sugars in 100 mL of wines [38], it can be deduced that the PAS 2022 wine has the highest sugar content, while AGR 2021 has the lowest one. This result completely fits the trend previously observed for viscosity and sound speed. Similarly, the highest density value was measured for PAS 2022 (0.9950–0.9951 g/mL), confirming its highest sugar content among all the analyzed wines. Indeed, sugars and other secondary compounds present in wine such as glycerol and tartaric acid can increase the density, while ethanol has the opposite effect by decreasing it [39]. The relationship between sound speed and ethanol content, sugar content (Brix values), and density was further evidenced by the high Pearson correlation coefficients. The strongest correlation was observed between the sound speed and Brix values (r = 0.9407), followed by the ethanol content (r = 0.8736) and density (r = 0.8315) (Figure S2).

**Figure 5:**
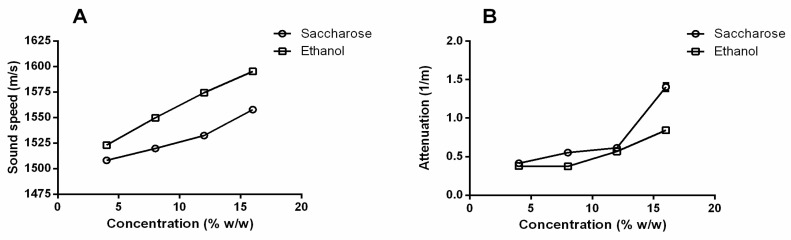
Variation in sound speed (m/s) (**A**) and variation in attenuation (1/m) (**B**) vs. concentration (% *w*/*w*) for standard solution of saccharose and ethanol at different concentrations (4, 8, 12, 16% *w*/*w*).

Standard solutions of saccharose and ethanol in water at the percentages of 4%, 8%, 12%, and 16% *w*/*w* were analyzed to further confirm that the ultrasound parameters (sound speed and attenuation) measured using the HR-US technique are mainly affected by the ethanol and sugar content, which are the two more abundant components in wine after water (Figure 5). The sound speed showed a linear increase as a function of both the saccharose and ethanol concentrations in the range investigated (4–16% *w*/*w*), differently from the attenuation parameter. Moreover, it has also emerged that the variation in sound speed over concentration is more pronounced in ethanol–water solutions than in water–saccharose solutions (Figure 5A). On the other hand, less marked differences were observed in the plots relating to the variation in the attenuation parameter (Figure 5B). Therefore, at least for sound speed, it seems that the ethanol content can have a predominant role over the sugar content in determining the ultrasound-responsive properties of wines, as previously reported by Lamberti et al. [22].

Notably, the measured absolute values from both attenuation (from ~0.5 to ~1.5 1/s) and sound speed (from ~1500 to 1600 m/s) were comparable to those measured for wines, confirming that ethanol and sugars are able to affect ultrasound measurements and are the main constituents on which differences in sound speed and attenuation in wines can be ascribed.

Ultrasonic spectroscopy’s limitations include sensitivity to dissolved CO_2_, which may distort compressibility and acoustic propagation. Small temperature drifts can affect ultrasound readings, necessitating strict thermal regulation. Additionally, compounds from barrel aging (e.g., tannins, polyphenols) could impact acoustic parameters due to complex matrix interactions. Manual data input into blockchain platforms introduces human error risk. Integrating Internet of Things (IoT)-based sensors for automated data acquisition and blockchain recording would enhance traceability and data integrity.

### 3.4. Soil Health Assessment Using the QBS-ar

Among soil fauna, soil microarthropods contribute significantly to organic matter decomposition, nutrient cycling, plant growth, and overall ecosystem functioning [40]. They are recognized as reliable indicators of soil health [26], capable of effectively assessing the sustainability of agricultural practices [27,41,42,43,44].

The application of the QBS-ar method to the vineyards in which the wines in our study were produced showed that the average total QBS-ar value obtained in the different vineyards was very high and similar between 2022 (184) and 2023 (181) (Table 4).

**Figure 6:**
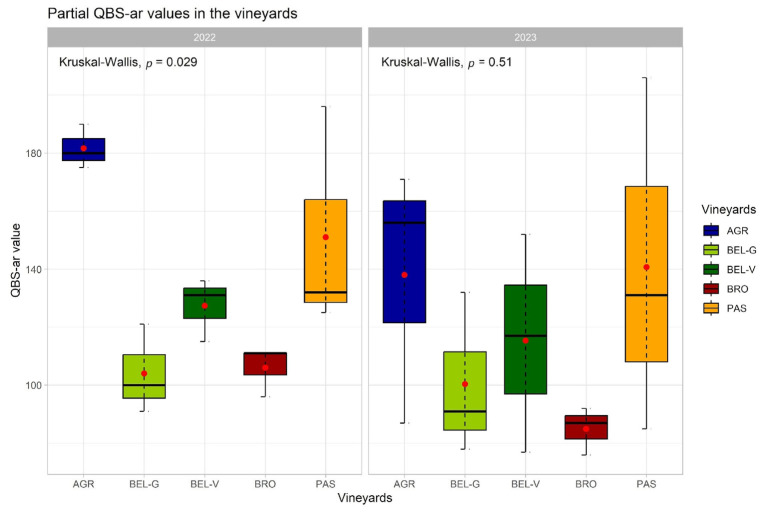
Boxplot of the distribution of the partial QBS-ar value obtained in each vineyard in 2022 and 2023. The package “ggplot2” was used to generate the plot [36].

The results show that a higher average partial QBS-ar value was found in 2022 (134) compared to 2023 (116), although the difference was not statistically significant. Likewise, no significant differences in the partial QBS-ar values were observed among the different vineyards for both years (Figure 5). In 2022, the vineyard of the Az. Agr. IIS G. Garibaldi showed a notably higher average partial QBS-ar value (182) than the other vineyards, particularly when compared to Cantina Belisario (BEL-G =104; BEL-V =127) and Brocani farm (106). Furthermore, the average partial QBS-ar value found in Az. Agr. Passacantando was high (151). Compared to 2022, 2023 exhibited greater homogeneity in values. Nonetheless, consistently with the previous year (2022), the vineyards of Az. Agr. Passacantando (141) and Az. Agr. IIS G. Garibaldi (138) showed higher values, while the vineyards of Az. Agr. Brocani (85) and the youngest (100) and oldest vineyard of Cantina Belisario (115) showed lower values (Figure 6).

The overall soil quality of the vineyards analyzed was excellent (Table 3). The total and average partial QBS-ar values registered in both years exceeded the threshold set for high biological quality in agricultural soils (93.7) [25,45]. In both 2022 and 2023, Az. Agr. Passacantando and Az. Agr. IIS G. Garibaldi vineyards recorded the highest QBS-ar values. This indicates that these farms have greater soil health, which can be attributed to their implementation of more sustainable agricultural practices. On the other hand, in both years, Brocani seemed to be the lowest-performing farm; in its case, more biodiversity-friendly practices could be implemented to enhance soil biodiversity. In fact, during the two years examined, this was the only farm that received a lower soil quality judgment, although it was good. The soil quality certificates for each year and each vineyard are available online at https://www.biocerto.it/certificati-biocerto (accessed on 26 May 2025).

### 3.5. Blockchain-Notarized Biocerto Certificate: Process Overview

The results obtained from soil and wine analyses enabled the creation of the Biocerto Certificate. Each production year (2021 and 2022) was linked to a unique Digital Certificate, accompanied by a corresponding QR code containing its identifier, which was subsequently printed on the product packaging. The Digital Certificate associated with each bottle is accessible via the Biocerto website (Biocerto.it). All analytical data were entered by operators into the Biocerto platform to generate a Digital Certificate specific to the analyzed wines and vineyard soils. A digital fingerprint of each Certificate was then computed using the SHA-256 cryptographic hash function. This fingerprint, represented as a unique alphanumeric string, serves as a tamper-evident identifier for the document. Any subsequent modification to the Certificate would result in a different hash value, thereby ensuring data integrity and traceability. The original hash was immutably recorded on the blockchain along with a timestamp, providing verifiable proof of the Certificate’s creation date. The blockchain ledger remains publicly accessible and independently verifiable at any time.

The functional diagram of the process for generating the Biocerto Certificate, notarized on the blockchain, is outlined below.

#### 3.5.1. Workflow Processes

1. System Identification and Registration
  Identification and registration of farms and associated land parcels involved in the certification process.
2. Sampling Calendar and Task Assignment
  Programming of a sampling schedule and delegation of specific tasks to qualified personnel.
3. Soil Sampling Procedure
  Execution of soil sampling according to predefined protocols. Each sample is accompanied by the following minimum metadata:
  ○ Farm name;
  ○ Land identification;
  ○ Geographic coordinates of the sampling location;
  ○ Date and time of collection;
  ○ Sample type;
  ○ Operator code (anonymized).
4. Analytical Procedures
  Laboratory analysis of collected soil samples (and, where applicable, wine samples), generating output files in standard formats (e.g., .txt, .csv).
5. Data Acquisition and System Integration
  ○ Ingestion of analytical output into the Biocerto platform.
  ○ Automatic association of analytical results with the corresponding sample metadata, including location and land origin.
6. Digital Notarization
  ○ Events related to sample collection and the associated analytical results are notarized via the Biocerto digital platform;
  ○ The platform interfaces with the Quadrans blockchain API to notarize each analytical record;
  ○ Each notarized event is timestamped and linked to a verifiable cryptographic record.
7. QR Code Generation and Product Labeling
  ○ Generation of unique QR codes corresponding to each certified product and its land of origin.
  ○ These QR codes are printable on product packaging and provide traceability for consumers.
8. Blockchain Query Functionality
  ○ End users and third parties can query the publicly accessible blockchain ledger to verify the authenticity and integrity of certificates.

#### 3.5.2. Certificate Composition and Blockchain Integration

The Biocerto Certificate is generated from comprehensive analyses of soil samples and wine products and includes the following components:

● Annual Certification and QR Code
  Each production year (e.g., 2021 and 2022) is assigned a unique Digital Certificate, which is associated with a corresponding QR code. This QR code can be printed on the final product, allowing consumers to verify the certificate via Biocerto.it.
● Data Entry and Certificate Generation
  Certified technicians and authorized operators input the complete set of analytical data into the Biocerto platform. This data is used to generate a verifiable Digital Certificate for the analyzed product.
● Cryptographic Hash Generation
  A cryptographic hash of the Certificate is calculated using the SHA-256 algorithm, resulting in a unique alphanumeric string (the “digital fingerprint”). This hash ensures the document’s integrity, as any modification would produce a different hash value.
● Blockchain Timestamping and Verification
  The hash of the original Certificate is immutably recorded on the Quadrans blockchain with an associated timestamp. This allows independent third-party verification of the Certificate’s authenticity and creation date. The blockchain ledger is publicly accessible and can be verified at any time.

#### 3.5.3. Infrastructure and Verification Capabilities

To support the notarization process, physical computing nodes are installed and connected to the Quadrans blockchain network. This infrastructure enables the rapid, reliable, and cost-effective registration of Certificates and laboratory reports, while also offering the advantage of maintaining a local copy of the blockchain ledger on site.

Key features include:

● Immutable Blockchain Registration
  The SHA-256 hash of each Certificate is permanently recorded on a public blockchain, ensuring traceability and tamper resistance.
● Document Authenticity Verification
  The QR code and blockchain record enable users to verify that the digital certificate matches the original. Verification includes access to metadata such as the following:
  ○ The docHash (document hash);
  ○ Blockchain registration timestamp;
  ○ Merkle Tree root and associated evidentiary data.

Overall, the system offers a robust and transparent framework for guaranteeing the integrity and traceability of certified wine and other agri-food products. Furthermore, we estimate that, once fully operational, the cost per bottle for productions under 100,000 bottles per year will be approximately EUR 0.01.

An example of Biocerto Certificate generation for the Brocani Farm and the “Verdicchio dei Castelli di Jesi Classico Superiore 2021 Ligami” is provided in the Supplementary Materials, along with a simplified diagram (Figure S3) illustrating the main phases of the process. Similarly, more information concerning the Blockchain Layer—Operational Specifications (Using Quadrans) and the cryptographic proof-of-concept have been included in the Supplementary Materials (Figures S4 and S5).

## 4. Conclusions

This study presented a method for developing an open prototype that safely incorporates unalterable data to certify the quality of wine, considering both its chemical–physical composition and the sustainability of its production. Various techniques, including ultrasound spectroscopy and the analysis of soil mesofauna, to evaluate soil health, have been applied. Ultrasound spectroscopy, especially in terms of the sound speed parameter, demonstrated a high sensitivity to discriminate among wines, at least comparable to that of the most common technique based on viscosity measurements. Indeed, as with viscosity, ultrasound speeds in wines seem to be mainly influenced by ethanol and sugar contents.

To summarize, the overall approach aligns with the growing consumer interest and demand for products with certified origins and sustainable production practices that do not compromise biodiversity preservation. We are confident that this model can be applied to other businesses and product types other than wine, and that it can be further implemented with additional analyses such as metabolomic fingerprinting, isotopic analysis [46], and further indicators of agroecosystem sustainability, including soil microbial communities and their enzymatic activity [47].

Preliminary estimates based on current service models for outsourced digital certification and environmental monitoring suggest that the adoption of this system could reduce fraud incidence by approximately 20–30% over time, particularly in markets exposed to mislabeling or adulteration. By integrating transparent traceability and authentication tools managed by a neutral third party, the platform has the potential to significantly boost consumer confidence, especially among buyers seeking certified sustainable and authentic products. For small-to-medium wineries, the economic burden is significantly reduced under a service-based model. In this scenario, wineries would only be responsible for submitting samples and relevant production data to an external entity that manages the platform and performs the QBS-ar and ultrasonic analyses. Estimated costs per winery would range from EUR 1000 to 2000 per year, depending on the sampling frequency, vineyard size, and service package. These figures are derived from analogous services currently offered by third-party certification bodies and laboratory analysis providers in the EU (e.g., eco-certification audits, remote sensing analysis, or enological profiling). Such a model minimizes internal infrastructure and personnel investment, making it more accessible for small producers. The moderate annual cost is likely to be offset by benefits such as improved access to high-value markets, enhanced brand reputation, and a measurable contribution to sustainability certification and fraud prevention.

Finally, we believe that collaboration with regulatory bodies such as the ICQRF—Ispettorato Centrale della tutela della Qualità e Repressione Frodi dei prodotti agroalimentari—Central Inspectorate for Quality Protection and Fraud Repression in Agri-food Products, a branch of the Italian Ministry of Agriculture responsible for ensuring the quality and compliance of agri-food products, including PDO wines—and EFSA (European Food Safety Authority) would be highly beneficial. Such cooperation could support pilot-scale validation and significantly accelerate the implementation and broader adoption of this approach.

## Figures and Tables

**Table 1:** Farms and vineyards involved in the study. In brackets the commercial name of the bottle of wine used through the study is reported.

Farm	Vineyard ID	Location	Geographical Coordinates		Wine	Berry Type
Az. Agr. I.I.S. G. Garibaldi	AGR	Macerata (MC)	43°17′8.83″ N	13°25′9.03″ E	Maceratino	White
Az. Agr. Brocani	BRO	Staffolo (AN)	43°27′5.89″ N	13°9′21.99″ E	Verdicchio dei Castelli di Jesi PDO Classico Superiore (Ligami)	White
Az. Agr. Passacantando	PAS	Tolentino (MC)	43°14′24.07″ N	13°17′14.09″ E	Sangiovese	Red
Cantina Belisario	BEL-G	Matelica (MC)	43°15′9.85″ N	12°59′14.87″ E	Verdicchio di Matelica PDO (Vigneti B)	White
Cantina Belisario	BEL-V	Matelica (MC)	43°15′7.95″ N	12°59′6.17″ E	Verdicchio di Matelica PDO (Vigneti B)	White

**Table 2:** Pedological origin of the vineyards involved in the study according to ASSAM, 2006.

Vineyard ID	Pedological Origin	Soil Typological Units (UTS)
AGR	5.3.8—Macerata and Corridonia hills	Haplic Calcisols (COR), Calcaric Regosols (SPO), Calcaric Cambisols ch-3 Vertic Cambisols (ETG-2)
BRO	5.3.2—Alta Vallesina hills	Haplic (Hypercalcic) Calcisols (FVT), Haplic Calcisols (BRN), Endosodi-Vertic Cambisols (CER)
PAS	5.3.3—Pitino and hills north of the Potenza river	Calcaric Regosols (SPO), Calcari-Hyposodic Regosols (PRD), Calcari-Endogleyic Cambisols (DIA)
BEL-G	3.3.4—Inland basin from Matelica to Candigliano	Calcaric Cambisols (LCS), Haplic Calcisols (PGL and BRN), Calcaric Regosols (MTV)
BEL-V	3.3.4—Inland basin from Matelica to Candigliano	Calcaric Cambisols (LCS), Haplic Calcisols (PGL and BRN), Calcaric Regosols (MTV)

**Table 3:** Refractive index, Brix, density values (g/mL), and ethanol content (% *v*/*v*) for the three wines produced in 2022 (BRO, BEL, and PAS) and the AGR produced in 2021.

	Refractive Index	Brix Values (°Bx)	Density (g/mL)	Ethanol Content (% *v*/*v*) *
BRO 2022	1.343–1.344	7–7.5	0.9895–0.9896	14
BEL 2022	1.343–1.344	7–7.0	0.9901–0.9902	13
PAS 2022	1.347–1.348	9–9.5	0.9950–0.9951	14
AGR 2021	1.341–1.342	6–6.5	0.9894–0.9895	11

* Ethanol content (% *v*/*v*) is from the manufacturer.

**Table 4:** Total QBS-arl values obtained in each vineyard in 2022 and 2023, and related soil quality judgments [35].

Vineyard	2022	Soil Quality Judgment	2023	Soil Quality Judgment
AGR	200	Excellent	192	Excellent
BRO	161	Excellent	143	Good
PAS	227	Excellent	212	Excellent
BEL-G	162	Excellent	163	Excellent
BEL-V	171	Excellent	193	Excellent

## Data Availability

Data are contained within the article.

## Supplementary Materials

The following supporting information can be downloaded at: https://www.mdpi.com/article/10.3390/s25113567/s1. Table S1: Average monthly temperature (°C) and precipitation (mm) in the years 2022 and 2023, obtained from 4 different meteorological stations, considering those closest and at similar altitudes to the sites under consideration; Figure S1: Dependence of ultrasound amplitude (mV) over frequency (MHz) in the range 1–16 MHz. Figure S2: Blockchain-Notarized Biocerto Certificate processes and example. Figure S2: Linear regression plots showing the correlation between sound speed and classical wine parameters (Brix, A; density, B; ethanol, C). Figure S3: Blockchain-notarized Biocerto Certificate processes and example. **A**: A schematic overview of the steps in the generation of the Blockchain-notarized Biocerto CertificatepProcesses; **B**: An example of Biocerto Certificate generation for the Brocani Farm and the “Verdicchio dei Castelli di Jesi Classico Superiore 2021 Ligami”. Figure S4: Details of the blockchain processes. A: Operational blockchain layer: (i) Data entry permissions, (ii) Hashing algorithm, (iii) Block interval, (iv) Consensus mechanism (PoA), and (v) On-chain/Off-chain data split. B: Flowchart of the end-to-end data flow from vineyard notebook to consumer-facing QR code. C: Graphic outline of the Biocerto Notarisation process (on Quadrans Blockchain). Figure S5: Cryptographic proof-of-concept.
