# Supplementary material for: A.A.A. Good Wines WANTED: Blockchain, Non-Destructive Ultrasonic Techniques and Soil Health Assessment for Wine Traceability
Source: Sensors (Basel). 2025 Jun 5;25(11):3567. doi: 10.3390/s25113567 (PMC12158379; doi:10.3390/s25113567)
Supplement: Supplementary file 1 [file sensors-25-03567-s001.zip › sensors-3522653-supplementary.pdf]

Supplementary materials

Table

Table S1. Average monthly temperature (°C) and precipitation (mm) in the years 2022 and 2023, obtained from 4 different meteorological stations, considering those closest and at similar altitudes to the sites under consideration.

Month	Temperature (°C)		Precipitations (mm)	
	2022	2023	2022	2023
1	5.5	6.7	22.1	134.2
2	8.3	6.9	67.0	47.1
3	6.8	11.2	21.0	71.6
4	11.6	11.3	51.8	77.0
5	19.1	16.0	29.6	196.9
6	24.9	21.3	38.9	179.0
7	26.1	26.3	63.5	26.5
8	24.2	24.1	28.7	61.8
9	19.5	21.1	202.6	36.8
10	17.2	19.4	5.6	32.6
11	11.1	12.1	114.5	143.2
12	9.5	9.0	86.4	36.4

Figures

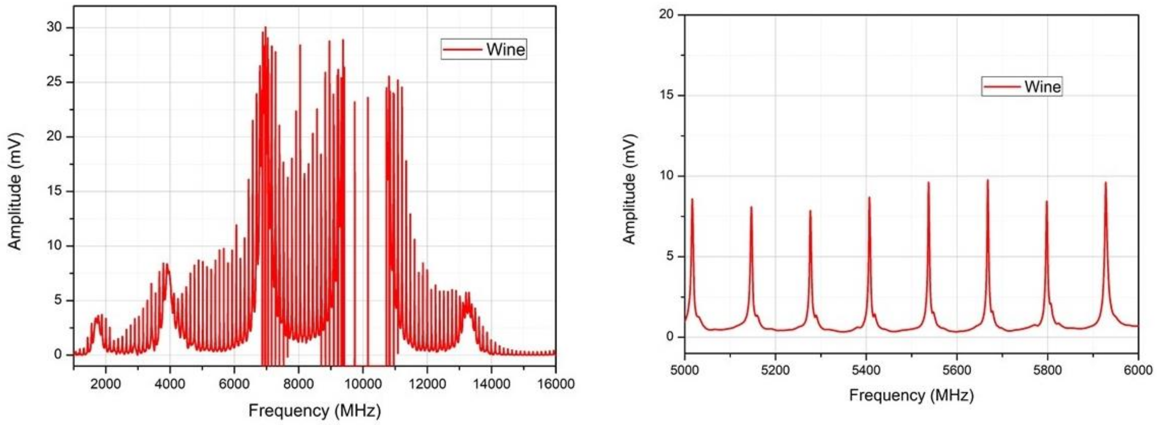

Fig. S1 - Dependence of ultrasound amplitude (mV) over Frequency (MHz) in the range 1-16 MHz.

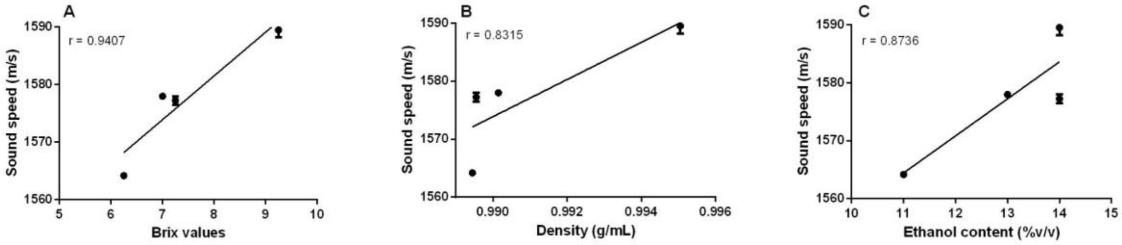

Fig. S2 - Linear regression plots showing the correlation between sound speed and classical wine parameters (Brix, A; density, B; ethanol, C).

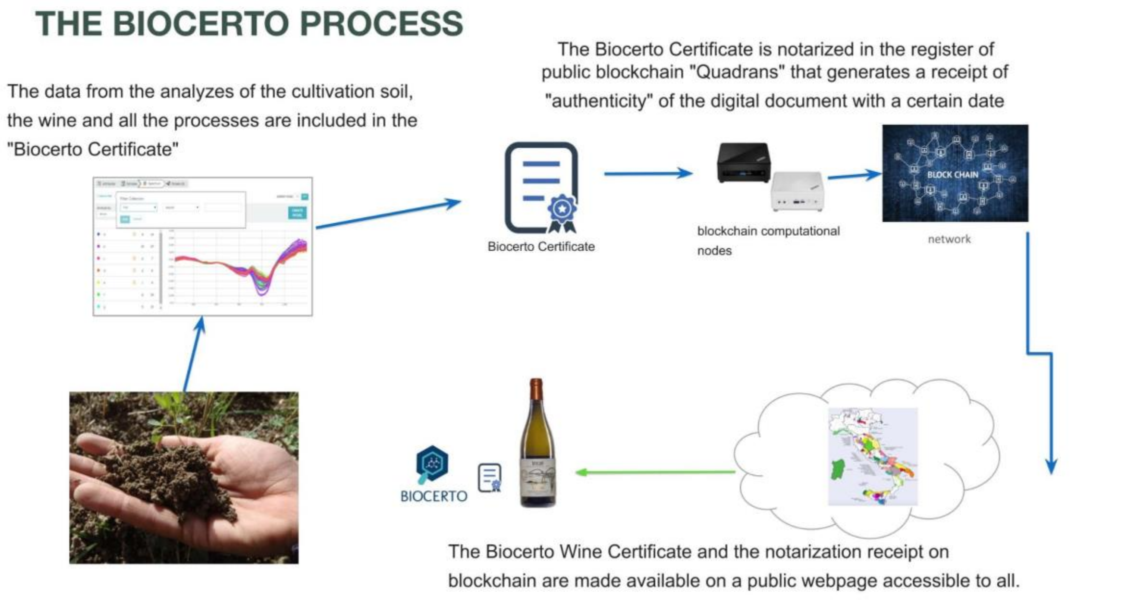

A

Example:

Biocerto Certificate Brocani Company Verdicchio di Jesi Classico Superiore 2021 Ligami

Block-data-time: 02/23/2024 15:07 UTC

Document Date:

docHash: e17786157987298c2937e314d1da024142c7179e6767d33ec5fef582140222c0

emailHash: 9a2646ce855498bc1eb2fb31d7e750cd8528a775aaada869b0bbfbbe4847b4b9

evidence: a8464de66b41044a4600920eb5f387cb3fc2725c9460beb00812b22217fa7fd1

The docHash string represents the cryptographic code of the Original Biocerto Certificate calculated with the SHA256 algorithm.

The "evidence" string is the alphanumeric cryptographic code combined with the "docHash" code, registered on the public blockchain and represents the information that must appear in the transaction monitor on the blockchain verification page, within the Merkel Tree, confirming the date of registration of the document.

B

Fig. S3 - Blockchain-Notarized Biocerto Certificate Processes and example. **A**: A schematic overview of the steps in the generation of the Blockchain-Notarized Biocerto Certificate Processes; **B**: An example of Biocerto Certificate generation for the Brocani Farm and the "Verdicchio dei Castelli di Jesi Classico Superiore 2021 Ligami".

Blockchain Layer – Operational Specifications (Using Quadrans):
(i) Data Entry Permissions
Public but controlled network:
- Any node can **read** the blockchain (*permissionless*),
- Only **authorized actors** (producers, certifiers, auditors) can **sign and write** data using their **registered private keys**, approved by Biocerto or the network governance.

- Decentralized structure with **wallets linked to real identities** (e.g., farms, certifying bodies, labs).

(ii) Hashing Algorithm
- SHA-256 – used to generate a unique digital fingerprint for documents (e.g., lab results, certifications).
- Only the hash is stored on-chain for immutability and verification.
- The full document is stored off-chain (e.g. Google Drive).

(iii) Block Interval
- **Approximately 5 seconds** on the Quadrans Mainnet – allows **near real-time recording**.
- Optimized for traceability and supply chain transparency.

(iv) Consensus Mechanism
- **Modified Proof of Authority (PoA):**
  - ❖ Consensus achieved through **validator masternodes** authorized by Quadrans governance.
  - ❖ Fast, low energy consumption,
  - ❖ Ideal for networks with **known and verified participants** (e.g., agri-food supply chains, public entities).

(v) On-chain / Off-chain Data Split

Data Type	Location	Reason
Certificate hashes, timestamps	**On-chain**	Immutability, verification
Original Certificate Pdf (Google Drive links)	**Off-chain**	Scalability, cost efficiency

A

End-to-End Flow – From Field to QR Code (With Quadrans)

```
flowchart TD
A[📒 Digital Vineyard Notebook (App/Web)] --> B[🔍 Review and Signature by Certifier]
B --> C[📄 Generate PDF Certificate]
C --> D1[🔐 SHA-256 Hash + Metadata]
D1 --> E[⛓ Transaction on Quadrans Blockchain]
C --> D2[📁 Upload Certificate to Google Drive]
E --> F[🖱 Generate QR Code with Hash + Link]
D2 --> F
F --> G[📱 QR Code Scan by Consumer]
G --> H[🌐 Biocerto Portal: Display Certificate + Origin Info]
```

B

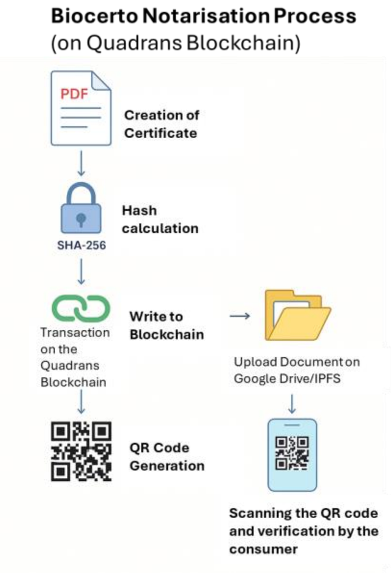

C

Fig. S4 - Details on the blockchain processes. **A:** Operational blockchain layer: (i) Data entry permissions, (ii) Hashing algorithm, (iii) Block interval, (iv) Consensus mechanism (PoA), and (v) On-chain/ Off-chain data split. **B:** Flowchart of the end-to-end data flow from vineyard notebook to consumer-facing QR code. **C:** Graphic outline of the Biocerto Notarisation Process (on Quadrans Blockchain).

Each relevant document (e.g., PDF certificate, soil analysis, cultivation log) is hashed (SHA-256) and inserted into a **Merkle tree**.
Typically, each production cycle generates:

- **1–5 hashes per batch** (certificates, analysis, QR codes, metadata),
- With ~100 batches per year, this results in **hundreds of Merkle leaves per winery annually**.

The use of a Merkle tree allows for:

- **Fast integrity verification** through Merkle proofs.

Collision Resistance
The system uses **SHA-256**, a hashing algorithm that provides:

- **High collision resistance** (negligible probability: 1 in 2^{128}),
- **Practical impossibility** of generating two different documents with the same hash,
- Guarantees **integrity, authenticity, and non-repudiation** of the information published on the **Quadrans blockchain**.

The **Quadrans blockchain is immutable**: once a hash is written, **it cannot be modified or deleted**.

To correct possible errors in certified data:

- An **append-only model** is used:
- A **new transaction** is published, referencing the erroneous one via its hash or ID,
- The new record includes a **semantic flag** (e.g., corretcs, Rev.2) in the associated metadata,
- The **Biocerto interface** (web portal) shows **only the latest valid version**, while preserving the entire history for auditing purposes.
  - **Immutability is guaranteed**
  - **GDPR compliance is ensured** — off-chain data can be updated or deleted, and on-chain data is pseudonymized

Fig. S5 - Cryptographic proof-of-concept.
